# BrainNeXt: novel lightweight CNN model for the automated detection of brain disorders using MRI images

**DOI:** 10.1007/s11571-025-10235-z

**Published:** 2025-03-22

**Authors:** Melahat Poyraz, Ahmet Kursad Poyraz, Yusuf Dogan, Selva Gunes, Hasan S. Mir, Jose Kunnel Paul, Prabal Datta Barua, Mehmet Baygin, Sengul Dogan, Turker Tuncer, Filippo Molinari, Rajendra Acharya

**Affiliations:** 1 Department of Radiology, Elazig Fethi Sekin City Hospital, Elazig, Turkey; 2 Department of Radiology, School of Medicine, Firat University, 23119 Elazig, Turkey; 3 Department of Electrical Engineering, American University of Sharjah, Sharjah, UAE; 4 Department of Neurology, Government Medical College, Thiruvananthapuram, Kerala India; 5 School of Business (Information System), University of Southern Queensland, Springfield, Australia; 6 Department of Computer Engineering, Engineering Faculty, Erzurum Technical University, Erzurum, Turkey; 7 Department of Digital Forensics Engineering, Technology Faculty, Firat University, Elazig, Turkey; 8 Department of Electronics and Telecommunications, Politecnico Di Torino, Turin, Italy; 9 School of Mathematics, Physics and Computing, University of Southern Queensland, Springfield, Australia

**Keywords:** BrainNeXt, MRI dataset, Deep feature engineering, INCA

## Abstract

The main aim of this study is to propose a novel convolutional neural network, named BrainNeXt, for the automated brain disorders detection using magnetic resonance images (MRI) images. Furthermore, we aim to investigate the performance of our proposed network on various medical applications. To achieve high/robust image classification performance, we gathered a new MRI dataset belonging to four classes: (1) Alzheimer's disease, (2) chronic ischemia, (3) multiple sclerosis, and (4) control. Inspired by ConvNeXt, we designed BrainNeXt as a lightweight classification model by incorporating the structural elements of the Swin Transformers Tiny model. By training our model on the collected dataset, a pretrained BrainNeXt model was obtained. Additionally, we have suggested a feature engineering (FE) approach based on the pretrained BrainNeXt, which extracted features from fixed-sized patches. To select the most discriminative/informative features, we employed the neighborhood component analysis selector in the feature selection phase. As the classifier for our patch-based FE approach, we utilized the support vector machine classifier. Our recommended BrainNeXt approach achieved an accuracy of 100% and 91.35% for training and validation. The recommended model obtained the test classification accuracy of 94.21%. To further improve the classification performance, we suggested a patch-based DFE approach, which achieved a test accuracy of 99.73%. The obtained results, surpassing 90% accuracy on the test dataset, demonstrate the effectiveness and high classification performance of the proposed models.

## Introduction

The brain is the most basic structure of the central nervous system (Garman 2011). It is one of the most complex and mysterious organs in the body (Petryński et al. 2023; Singh et al. 2023). This organ works through neural networks and neural communication (Majhi et al. 2019). The nerve cells in the brain transmit electrical and chemical signals to the various organs of the body to control their working patterns (Levitan and Kaczmarek 2002). In this way, sensory, cognitive, emotional and physical abilities are controlled, which are basic human components (Tan and Nijholt 2010). These capabilities enable individuals to engage with the external world and interact with their surroundings. Structurally, the brain consists of four lobes, namely the frontal, parietal, temporal, and occipital lobes (Lu et al. 2022). Ongoing investigations indicate that each lobe is linked to distinct activities. For instance, the frontal lobe plays a role in cognitive functions (Ventura‐Campos et al. 2022) such as problem-solving, behavioral control, and personality expression, whereas the parietal lobe facilitates environmental awareness and spatial orientation (Bruner et al. 2023). Similarly, the temporal lobe is involved in the control of skills related to smell and hearing (Thalbourne et al. 2003). The occipital lobe, at the back of the brain, generally controls visual processing and vision-related functions (Dong et al. 2012). As might be expected, damage of these lobes affects the functions of the relevant lobe and causes different symptoms and diseases in individuals. As a result of brain damage, diseases such as AD, Parkinson's disease (PD), schizophrenia and epilepsy can occur, severely impacting an individual's quality of life and, in advanced stages, resulting in mortality (Emard et al. 1995; Németh et al. 2006; Welsh 2001).

Early diagnosis plays a key role in improving the people’s quality of life with brain disorders (Paulsen et al. 2013). Early detection can potentially halt, slow or treat disease progression. Currently, cutting-edge artificial intelligence (AI) technologies offer compelling solutions in this area. Recent advances, particularly in signal and image processing, have enabled the early diagnosis of many diseases (Saqib et al. 2023). An extensive review of the literature reveals remarkable accuracy in the classification and interpretation of medical images, including but not limited to magnetic resonance images (MRI), computed tomography (CT) scans and X-rays (Chakraborty and Mali 2023). This paper presents a novel, lightweight methodology called “BrainNeXt” for the initial diagnosis of selected brain-related disorders. We introduced a new generation CNN model to automate the classification of AD, MS, and CI, and this model is termed BrainNeXt. In addition, a BrainNeXt-based deep FE approach is suggested. To develop this deep FE model, local features are extracted using fixed-size patches inspired by vision transformers.

### Literature review

Medical image classification using machine learning methods is one of the most frequently studied topics in the literature (Celard et al. 2023). In this study, AD, MS and CI were classified using MRI images. In the review of the literature, it was noted that there was no study in which these diseases were considered as a whole. we explore the distinctive clinical presentations, temporal profiles, and radiological characteristics of two prominent diseases: MS and AD. AD is recognized as an inflammatory disorder that primarily manifests through recurrent neurological deficits in young patients. Notably, it is characterized by distinctive white matter lesions with a predilection for the pericallosal white matter, juxtacortical region, brainstem, and spinal cord. On the other hand, AD typically exhibits a more gradual cognitive decline, initially affecting episodic memory and subsequently extending to other cognitive domains. This progression is accompanied by medial temporal atrophy and the deposition of beta-amyloid and phosphorylated tau, which can be effectively demonstrated using PET imaging. For this reason, the literature review presented in this study consists of studies including the classification of related diseases. Some recent studies on machine learning-based classification of the diseases considered in this study are summarized in Table 1.

**Table 1:** State-of-the-art automatic brain disorder detection

Author(s)	Data type	Data	Method	Highlights
Murugan et al. (2021)	MRI	MiD-896 MoD-64 vMD-2240 CO-3200	Data augmentation, Custom-designed CNN (DEMNET)	The data is balanced with data augmentation AD is classified according to their level The computational complexity is high Classification performance is high (> 95%)
Kaplan et al. (2021)	MRI	AD-569 CO-601	Feed-forward local phase quantization network	Three datasets were used for validation Binary classification was performed (> 99%)
Garcia et al. (2023)	MRI	AD-60 MCI-30 PD-70 MS-30 CO-150	Spatially informed Bayesian neural network, custom-designed CNN	A dataset with 5 classes was used Neurodegenerative diseases are analyzed The computational complexity is high Accuracy is relatively low (= 83%)
Faisal et al. (2023)	GRFs	HD-20 PD-15 ALS-13 CO-16	Custom-designed CNN (NDDNet)	A dataset with 4 classes was used Neurodegenerative diseases are analyzed The computational complexity is high While successful results were obtained in binary classification, relatively low accuracy was achieved in multiclass classification (~ 83%)
Amooei et al. (2023)	GRFs	HD-20 PD-15 ALS-13 CO-16	Data augmentation, Spectrogram transformation, wavelet transform, CNN-LSTM	A dataset with 4 classes was used Neurodegenerative diseases are analyzed The computational complexity is high Accuracy is high (> 99%)
Dogan et al. (2023)	EEG	AD-12 CO-11	Primate brain pattern, TQWT, iterative majority voting, kNN	The computational complexity is linear Dataset is relatively small Binary classification was performed (~ 92%)
Alsharabi et al. (2023)	MRI	AD-358 PD-423 CO-229	AlexNet-based quantum transfer learning method	Neurodegenerative diseases are analyzed The computational complexity is high Binary classification was performed (> = 96%)
Pandian and Udhayakumar (2023)	MRI	CIS-87 RRMS-87 PPMS-87 SPMS-87 CO-87	Chaotic Leader-Selective Particle Swarm Optimization, Hybrid deep CNN	Two datasets were used for validation The levels of MS disease are categorized Classification performance is high (> 98%)
Acar et al. (2022)	MRI	MS-971 CO-971	Data augmentation, Custom designed CNN	The data is balanced with data augmentation The computational complexity is high Binary classification was performed (= 98%)
Balasundram et al. (2023)	MRI	MiD-896 MoD-64 vMD-2240 CO-3200	Custom designed CNN	Two datasets were used for validation AD is classified according to their level The computational complexity is high Classification performance is relatively high (> 94%)
Kaplan et al. (2023)	CT MRI	AD-569 CO-601	Patch division, LBP, LPQ and HOG, NCA, SVM	Two datasets were used for validation The computational complexity is linear Binary classification was performed (= 100%)

*MCI* mild cognitive impairment, *CO* control, *CIS* clinically isolated syndrome, *RRMS* relapsing–remitting MS, *PPMS* primary progressive MS, *SPMS* secondary progressive MS, *HD* Huntington’s disease, *ALS* amyotrophic lateral sclerosis, *MiD* mild demented, *MoD* moderate demented, *vMD* very mild demented, *GRF* ground reaction force

An examination of the literature studies listed in Table 1 shows that both signal processing (Amooei et al. 2023; Dogan et al. 2023) and image processing/deep learning (DL) methods (Balasundaram et al. 2023; Kaplan et al. 2023) are used in the diagnosis of brain-related diseases. Some of these studies aim to determine the severity of the disease (Balasundaram et al. 2023; Murugan et al. 2021; Pandian and Udhayakumar 2023), while others only make a classification between healthy and diseased (Acar et al. 2022; Alsharabi et al. 2023; Payares‐Garcia et al. 2023). In addition, some studies include different types of diseases together (Amooei et al. 2023; Faisal et al. 2023). However, these studies generally aim to classify neurodegenerative diseases (Payares‐Garcia et al. 2023). In our study, in addition to AD and MS, which are neurodegenerative diseases (MS is primarily characterized as an inflammatory disorder, although it also involves a component of neurodegeneration), chronic ischemia is also included in the dataset and classified. Chronic ischemia is not a neurodegenerative disease. Ischemia is a condition of reduced blood flow to tissues and is a condition that can be seen in any tissue in the body (Walter 2022; Zhang et al. 2019). Among the elderly population, ischemia is a frequently observed disorder, often resulting from uncontrolled hypertension, diabetes, dyslipidemia, and various other genetic predisposing conditions (Das et al. 2023). Early diagnosis and treatment of chronic ischemia is therefore important to increase the quality of the life.

### Motivations and our models

We have three essential motivations and these motivations are defined as follows:

Firstly, our motivation stems from the need to curate a comprehensive dataset consisting of MR images, specifically targeting cases of AD, MS, and chronic ischemia disorders. Our goal is to thoroughly investigate the classification capabilities of our proposed methods on this carefully collected dataset.

Secondly, we are driven by the objective of proposing a novel DL network. In the 2020s, transformer models have demonstrated remarkable performance in classification tasks, surpassing the commonly used convolutional neural networks (CNNs) such as ResNet. Thus, to keep pace with these advancements, a new generation of competitive CNNs has emerged, one of which is ConvNeXt (Liu et al. 2022). Through our research, we aim to present the effectiveness and potential of this new CNN model.

Lastly, we are motivated to introduce a fresh approach to deep FE, incorporating transfer learning and fixed-size patch division. Leveraging the pretrained BrainNeXt model, we extract deep features from each patch using the training signals. Our objective is to showcase the high classification capability offered by this novel deep FE approach.

We suggest both a new generation CNN and deep FE models. BrainNeXt shares similarities with ConvNeXt, albeit with a different configuration. ConvNeXt introduces a novel block structure employing 7 × 7 and 1 × 1 convolutions to create an inverted bottleneck, layer normalization, and the Gaussian Error Linear Unit (GELU) activation function. In contrast, BrainNeXt adopts 7 × 7 and 1 × 1 convolutions to form an inverted bottleneck and employs leaky rectified linear units (ReLU), similar to DarkNet, along with batch normalization. Additionally, we employ maximum pooling and concatenation layers to construct the proposed BrainNeXt architecture.

Transformers have commonly utilized fixed-size patches to extract image features. Building upon this attribute of transformers, we propose an exemplary deep-FE approach. Our model divides the input image into patches of size 32 × 32. We train the proposed BrainNeXt using the training dataset and utilize the global average pooling (GAP) layer of the pretrained BrainNeXt to extract features from both patches and the entire image. Neighborhood Component Analysis (NCA) (Goldberger et al. 2004) feature selector (FS) has been applied. The Support Vector Machine (SVM) (Vapnik 1998) has been used and the chosen features have been utilized as the input of the used SVM.

In summary, this paper presents a new generation of CNN and deep FE models. By conducting extensive experiments and analyses, we demonstrate the classification capability and potential applications of these proposed models.

### Innovations and contributions

#### Innovations

- To the best of our knowledge, our collected dataset is the first to include images related to MS, AD, and chronic ischemia. Hence the dataset is used for the automated classification of MS, AD and ischemia classes. A novel BrainNeXt model developed using CNN architecture. The proposed BrainNeXt achieved high classification performance for input MRI images. Additionally, BrainNeXt is a lightweight CNN since it contains fewer than 10 million (about 8.9 million) learnable parameters.
- We introduced a new generation deep FE approach. It demonstrated a high classification performance with linear time complexity (computationally efficient). Also, the BrainNeXt-based deep FE approach has improved the test classification accuracy of BrainNeXt.

#### Contributions

- We collected the MRI dataset belonging to MS, AD, and chronic ischemia classes. We achieved the classification accuracy of over 90% on the collected MRI image dataset using our proposed BrainNeXt model.
- This study presents a novel lightweight DL approach called BrainNeXt. It achieved high classification performance with fewer learnable parameters. Additionally, we demonstrated the explainable artificial intelligence (XAI) capabilities of BrainNeXt by highlighting important regions in each class to provide confidence to clinicians.
- We suggested a patch-based deep FE approach called BrainNeXt-based deep FE approach to enhance the test classification performance of the recommended BrainNeXt.

## Dataset

**Fig. 1:**
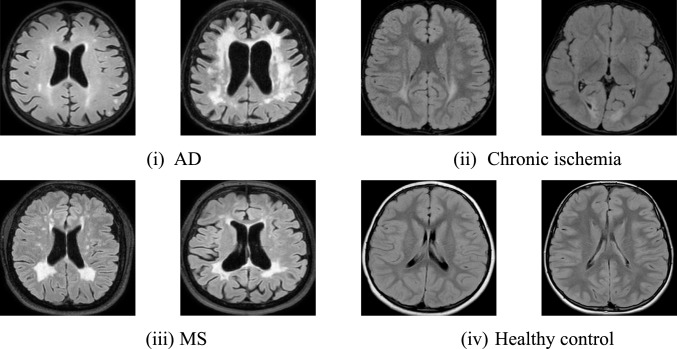
Sample images used from the dataset used

The dataset used in this work was obtained retrospectively and consists of four classes: (1 AD, (2) chronic ischemia, (3) MS, and (4) control. Four radiologists meticulously assessed and approved the collected images. The MRI scans were conducted between 01/01/2016 and 31/12/2023, with the radiologists carefully selecting the most relevant images. The dataset comprised of T2-weighted FLAIR images collected from 2,100 Turkish and Arabic participants aged between 32 and 89. This dataset includes 1269 female and 831 male MRIs collected from a medical center. The sample collected images are shown in Fig. 1.

The attributes of this dataset have been tabulated in Table 2.

**Table 2:** Details of the collected dataset used

No	Class	Number of images		Number of participants	
		Train	Test	Train	Test
1	AD	900	296	448	148
2	Chronic ischemia	302	84	148	43
3	MS	897	339	440	167
4	Control	1027	403	514	192
Total		3126	1122	1550	550

## Our proposals

In this work, we introduce BrainNeXt, a novel framework designed to address the challenges associated with brain-related tasks. Furthermore, we propose an innovative FE approach, built upon the foundations of BrainNeXt. In this section, the details of the suggested deep models have been explained.

### BrainNeXt

**Fig. 2:**
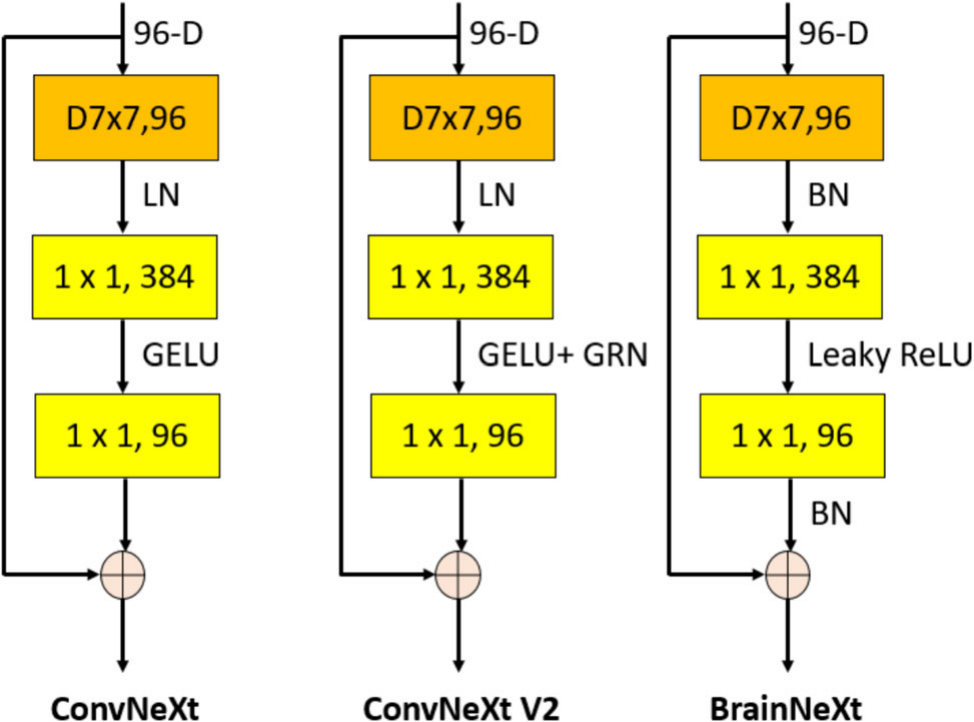
Block designs of the ConvNeXt, ConvNeXt V2 and the proposed BrainNeXt. **D7 × 7: Depthwise convolution with 7 × 7 sized kernel, LN: Layer Normalization, GELU: Gaussian Error Linear Unit, BN: Batch Normalization, ReLU: Rectified Linear Unit

In this research, we introduce BrainNeXt, a novel generation convolutional neural network (CNN) specifically tailored for brain-related tasks. To ensure its efficiency, we leverage the lightweight structure of the ConvNeXtV2 model (Woo et al. 2023). Within the BrainNeXt framework, we employ an inverted bottleneck design for convolutions, employing 7 × 7 and 1 × 1 convolutional kernels. Additionally, maximum pooling with a filter size of 3 × 3 and stride of 2 × 2 is utilized for compression. To augment the number of filters, we leverage depth concatenation. Notably, we have made modifications to the ConvNeXt block, resulting in the creation of our customized ConNeXt block and ConvNeXt V2 block. Figure 2 provides a visual representation of these blocks.

By using the above block (similar to ConvNeXt blocks), we have created the presented BrainNeXt. The details of the presented BrainNeXt are outlined in Table 3.

**Table 3:** Details of the BrainNeXt approach

Layer	Input size	Operation	Output size
Stem	224 × 224	4 × 4, 96, stride: 4	56 × 56
Layer 1	56 × 56	$$\left[\begin{array}{c}d7\times 7, 96\\ 1\times 1, 384\\ 1\times 1, 96\end{array}\right]\times 2$$	28 × 28
Layer 2	28 × 28	$$\left[\begin{array}{c}d7\times 7, 192\\ 1\times 1, 768\\ 1\times 1, 192\end{array}\right]\times 2$$	14 × 14
Layer 3	14 × 14	$$\left[\begin{array}{c}d7\times 7, 384\\ 1\times 1, 1536\\ 1\times 1, 384\end{array}\right]\times 6$$	7 × 7
Layer 4	7 × 7	$$\left[\begin{array}{c}d7\times 7, 768\\ 1\times 1, 3072\\ 1\times 1, 768\end{array}\right]\times 2$$	7 × 7
Output size	7 × 7	Global average pooling, fully connected layer, softmax	Number of classes
Number of learnable parameters			8.9 Millions

**Fig. 3:**
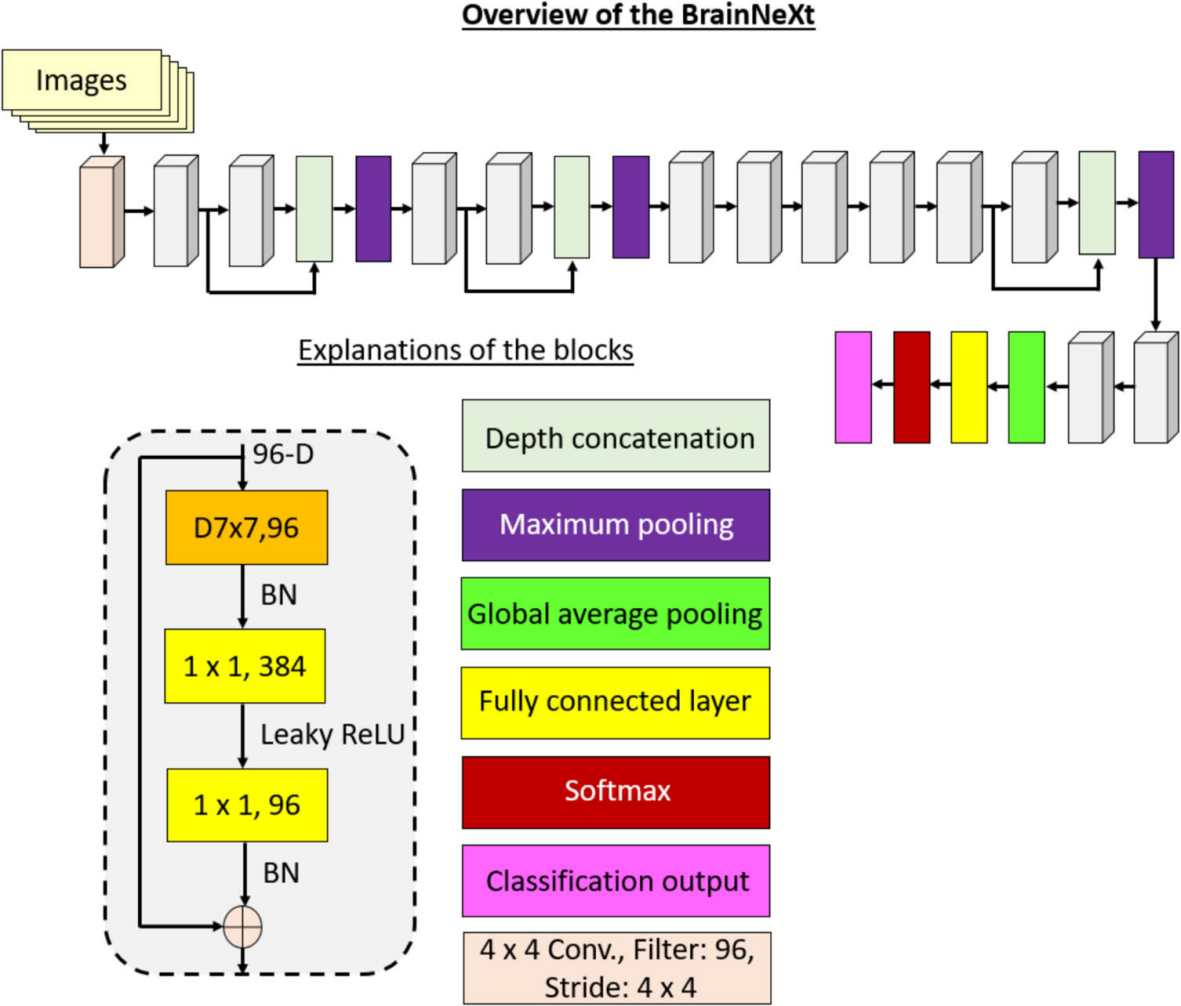
Graphical overview of the presented BrainNeXt

To better explain the proposed BrainNeXt, the graphical explanation is given in Fig. 3.

As depicted in Fig. 3, the presented BrainNeXt model incorporates several key components. Firstly, we employ a ConvNeXt-like block, which exhibits similarities to the ConvNeXt architecture. Secondly, the DarkNet activation function, utilizing leaky ReLU, is employed to enhance the model's representational capabilities. Finally, the structural elements of the swin transformer or ConvNeXt V2 tiny are incorporated into the model's design.

It is worth noting that the presented BrainNeXt model possesses approximately 8.9 million trainable parameters, rendering it a lightweight convolutional neural network (CNN). This characteristic allows for efficient training and inference while maintaining competitive performance.

### BrainNeXt-based exemplar FE approach

**Fig. 4:**
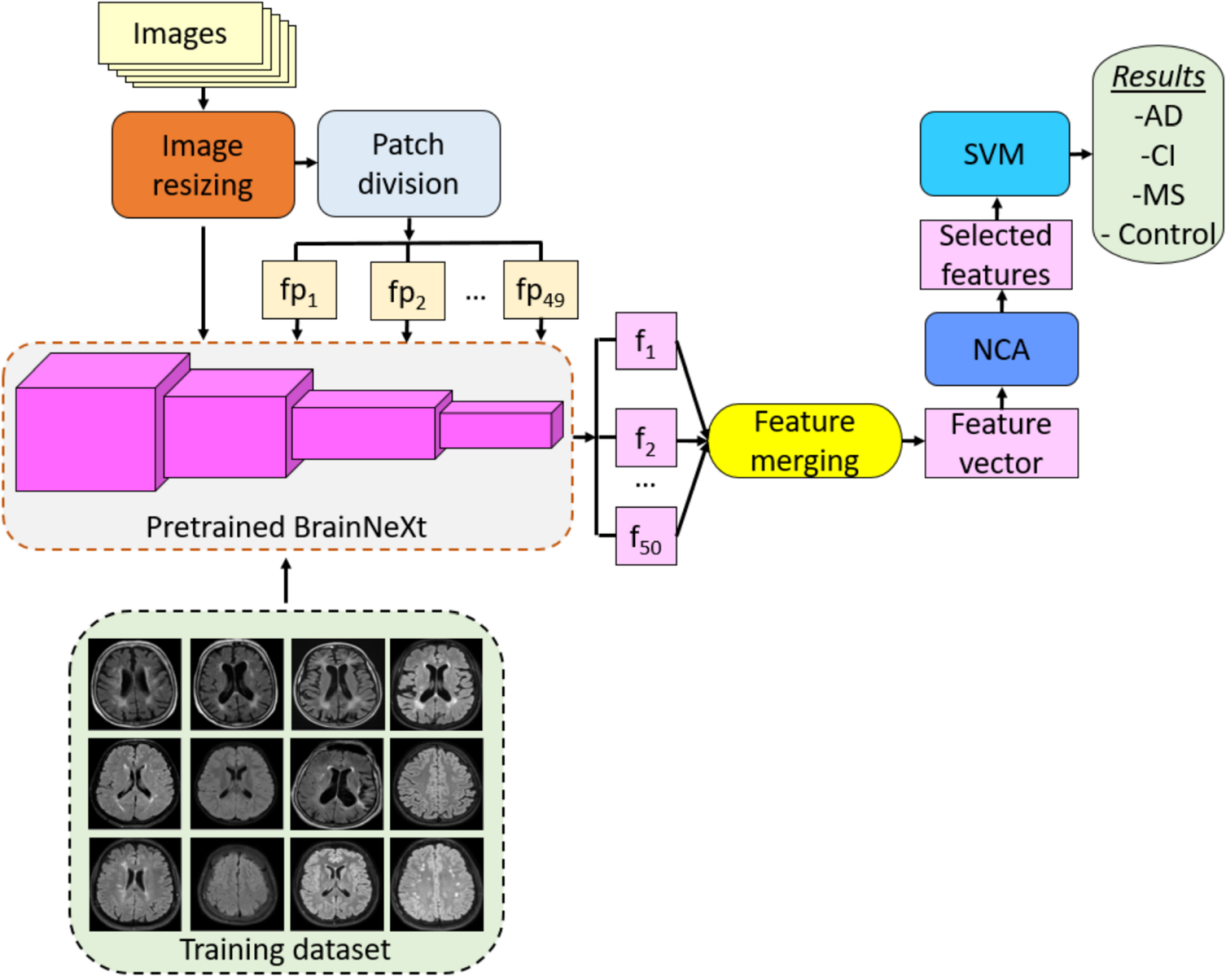
Schematic depiction of the proposed BrainNeXt-based FE architecture. **fp: fixed-size patch, f: feature vector, CI: chronic ischemia

To enhance the FE process, we have presented an exemplar (fixed-size patch) model built upon the pre-trained BrainNeXt network. The suggested FE approach, which leverages the capabilities of the presented BrainNeXt, is illustrated in Fig. 4. The diagram provides a high-level overview of the key components and their interactions within the FE approach, showcasing its efficacy in extracting informative features from the data.

As depicted in Fig. 4, our suggested approach consists of three fundamental phases: (i) exemplar deep feature extraction (FEX), (ii) FS, and (iii) classification.

During the FEX phase, we resize the input image to a size of 224 × 224 and create fixed-size patches with dimensions of 32 × 32. This process results in the creation of 49 patches (${\left(\frac{224}{32}\right)}^{2}$). We utilize the global average pooling layer of the proposed BrainNeXt network as the FEX, generating features from both the patches and the raw image. As a result, we obtain a total of 50 feature vectors (49 patches + 1 raw image). Finally, these 50 feature vectors are merged to create a single final feature vector.

The raw image (224 × 224) provides a holistic view of the entire MRI, enabling the model to capture global features, such as general structural patterns and large-scale abnormalities. In contrast, fixed-size patches (32 × 32) focus on localized regions of the image, allowing the model to detect fine-grained details, such as small lesions or subtle abnormalities that may be overlooked in the global context. The global average pooling layer in BrainNeXt extracts representative features from both the patches and the raw image, processing these inputs uniformly to ensure consistency in FEX. Combining features from these two perspectives (global and local) improved the classification ability of the approach. Additionally, using 32 × 32 patches reduces the time complexity of the FEX process while maintaining high performance. Hence, the patch size of 32 × 32 provided the best results.

To select the most informative features from the generated feature vector, we employ NCA (Goldberger et al. 2004). NCA utilizes a distance metric, such as L1-norm/Manhattan distance, to compute the weights of the features. It employs an optimizer, such as stochastic gradient descent (SGD), and generates non-negative features. NCA can be viewed as a FS variant of the k-nearest neighbors (kNN) (Peterson 2009) and is known to enhance the classification capabilities of the classifiers. Given its effectiveness, NCA is a widely recognized and popular FS within the field of FE.

To perform the classification task, we apply SVM (Vapnik 1998) to the selected features obtained from NCA. The following steps outline the methodology employed in this approach.

*Step 1:* Resize the image to 224 × 224.

*Step 2:* Apply patch division operator and create 49 patches and the size of each patch is 32 × 32.

1 $$\begin{aligned} & fp^{h} \left( {ii,jj,k} \right) = Im\left( {i + ii - 1,j + jj - 1,k} \right),i \in \left\{ {1,33,65 \ldots ,193} \right\}, k \in \left\{ {1,2,3} \right\} \\ & j \in \left\{ {1,33,65 \ldots ,193} \right\}, ii \in \left\{ {1,2, \ldots ,32} \right\},jj \in \left\{ {1,2, \ldots ,32} \right\}, h \in \left\{ {1,2, \ldots ,49} \right\} \\ \end{aligned}$$

Herein, $fp$ defines fixed-size patch and $Im$ is image. The above equation mathematically defines the patch division process.

*Step 3:* Extract features by using global average pooling layer of the trained BrainNeXt.

2 $$fv_{1} = BrainNeXt\left( {Im,GAP} \right)$$

3 $$fv_{h + 1} = BrainNeXt\left( {fp^{h} ,GAP} \right)$$

where $fv$ defines the feature vector and the proposed BrainNeXt defines as a function. The parameters of the $BrainNeXt(.,.)$ function is the used input and the used layer for FEX. In this step, 50 feature vectors have been created. As can be noted in Table 2, the length of each feature vector is 768.

*Step 4:* Construct final feature vector by merging the generated 50 feature vector.

4 $$F\left(q+768\times \left(t-1\right)\right)=f{v}_{t}\left(q\right), t\in \left\{\text{1,2},\dots ,50\right\}, q\in \left\{\text{1,2},\dots ,768\right\}$$

Herein, $F$ is the feature vector with a length of 38,400 (= 768 × 50).

*Step 5:* Identify the most informative 100 features out of the generated 38,400 features.

5 $$index=NCA(F,y)$$

6 $$s\left(w,r\right)=F\left(w,index\left(r\right)\right), w\in \left\{\text{1,2},\dots ,n\right\},r\in \left\{\text{1,2},\dots ,100\right\}$$

where $s$ defines the selected feature vector, $NCA(.,.)$ implies the NCA FS, $index$ represented the qualified indexes of the features by generating NCA, $y$ is actual output and $n$ defines the number of the observation (MRIs).

*Step 6:* Classify the selected features by deploying SVM.

The given six steps above have been defined the suggested FE approach.

## Experimental results

In this research, we have presented two image classification models: the presented BrainNeXt model and the BrainNeXt-based FE approach. To implement these models, we utilized the MATLAB programming environment, specifically leveraging the MATLAB deep network designer tool. Below, we provide the details of the parameters employed in these proposed models.

**Fig. 5:**
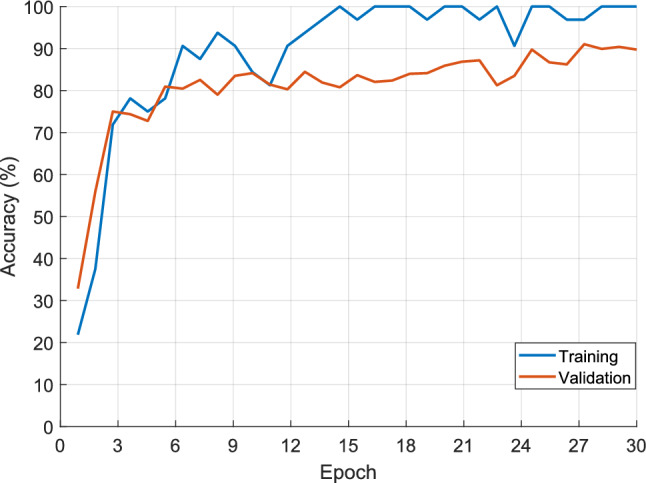
Training and validation curves obtained for the suggested approach

To obtain classification results, we trained the BrainNeXt model using the designated train dataset. The training process involved the utilization of parameters outlined in Table 4. Throughout the training phase, we monitored and recorded the performance metrics on both the training and validation datasets. The graphical representations of these results have been observed in Fig. 5, providing valuable insights into the approach's progress and performance.

**Table 4:** Hyperparameters used for the proposed models

Model	Parameters	Value
BrainNeXt	Split ratio	Training: 70%, validation: 30%
	Solver	SGDM
	Validation Frequency	50
	Epoch	30
	Mini Batch Size	32
	L2 Regularization	10^–4^
	Momentum	0.1
	Initial learning rate	0.01
BrainNeXt-based FE	Size of the patch	32 × 32
	FEX function	Global average pooling layer
	FEX	50 feature vectors (the length of each feature vector is 768) are extracted by deploying the global average pooling layer, patches and the MRI
	Feature merging	The length of the final feature vector is 38,400
	FS	The most informative 100 features are selected
	Classification	SVM: Kernel function: Cubic, C value (box constraint level): 1, coding: One-vs-all, validation: tenfold cross-validation (CV)

**Fig. 6:**
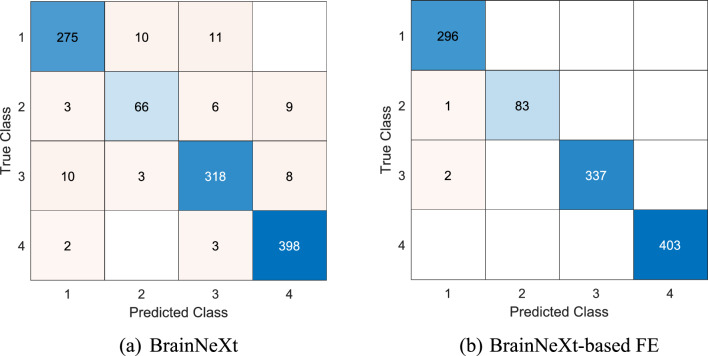
Confusion matrices obtained for the two models. **AD, 2: Chronic ischemia, 3: MS, 4: Control

The final validation accuracy for the trained BrainNeXt model was computed as 91.35%. We proceeded to evaluate its performance on the test dataset. Additionally, we employed the proposed BrainNeXt-based approach on the test images during the second phase of evaluation. To assess the quality of the test results, we employed various metrics, including classification accuracy, precision, recall, and F1-score. These metrics were computed by extracting the confusion matrices, which provide valuable insights into the model's performance for each class. The confusion matrices, highlighting the distribution of predictions and ground truth labels, are illustrated in Fig. 6.

Per the Fig. 6, the computed evaluation metrics have been summarized in Table 5.

**Table 5:** Results (%) of the suggested deep models for the test dataset

Model	Class	Accuracy	Precision	Recall	F1-score
BrainNeXt	AD	–	94.83	92.91	93.86
	CI	–	83.54	78.57	80.98
	MS	–	94.08	93.81	93.94
	Control	–	95.90	98.76	97.91
	Overall	94.21	92.09	91.01	91.52
BrainNeXt-based FE	AD	–	99	100	99.50
	CI	–	100	98.81	99.40
	MS	–	100	99.41	99.70
	Control	–	100	100	100
	Overall	99.73	99.75	99.55	99.65

Table 5 presents the comprehensive class-wise and overall results of both proposed models. The presented BrainNeXt achieved a commendable test accuracy of 94.21%, while the exemplar FE approach attained an impressive test accuracy of 99.73%. These outcomes unequivocally demonstrate the effectiveness of our suggested BrainNeXt approach for MRI classification, showcasing the utility of patch-based transfer learning.

Furthermore, we computed the total number of learnable parameters in the proposed BrainNeXt approach, which amounts to 8.9 million. This result underscores the lightweight nature of the model, affirming its status as a compact CNN.

## Discussions

**Fig. 7:**
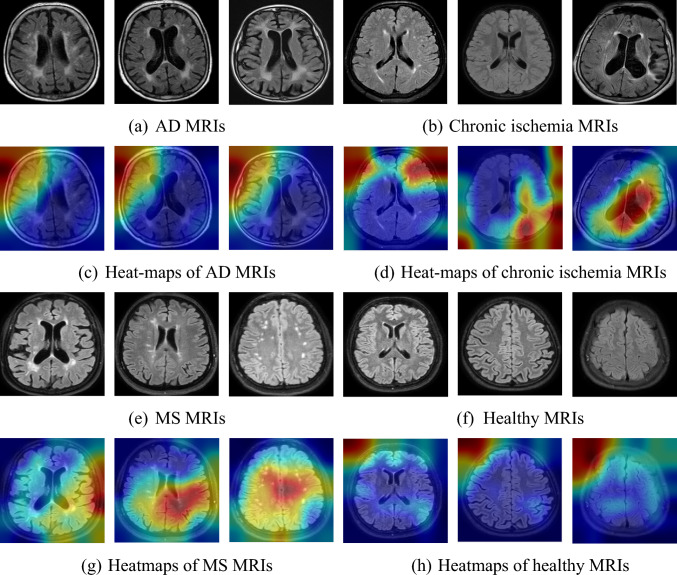
Grad-CAM results obtained for sample MRI images

In our approach, we collected a novel MRI dataset comprising four categories. The purpose of collecting this dataset was to augment the exposure and comprehensiveness of our suggested approach. Inspired by the ConvNeXt architecture, we have developed the BrainNeXt model to enhance the classification performance in this research. To provide further insights into the model's classification efficacy, we incorporated an explainable method known as a gradient-weighted class activation map (Grad-CAM). By deploying Grad-CAM, we generated heat maps for a selection of sample images, illustrating the regions of interest and highlighting the model's attention. These informative heat map images have been illustrated in Fig. 7, enabling a deeper understanding of the BrainNeXt model's classification capabilities using an explainable method.

Figure 7 showcased the ability of the presented BrainNeXt to focus on distinct regions of interest (ROI) for each class. Notably, the proposed BrainNeXt model accurately identifies and emphasizes abnormal areas in the case of disorders while emphasizing corner regions to extract distinguishing features from healthy MRIs. These interpretable findings, as showcased in Fig. 6, served as the foundation for our proposal of a patch-based FE approach. By extracting informative features from the patches, we successfully developed a patch-based deep FE approach, which exhibits a test accuracy that surpasses BrainNeXt by 5.52% (99.73%—94.21%).

The results obtained using XAI are given below:

- Figure 7c indicates the ability of our model to focus on regions related to AD pathology, such as cortical atrophy and ventricular enlargement.
- Figure 7d highlights ischemic lesions and regions of reduced perfusion.
- Figure 7g demonstrates white matter lesions of MS cases, particularly in periventricular regions.
- Figure 7h shows the corners (black areas) and highlights the absence of abnormalities.

**Fig. 8:**
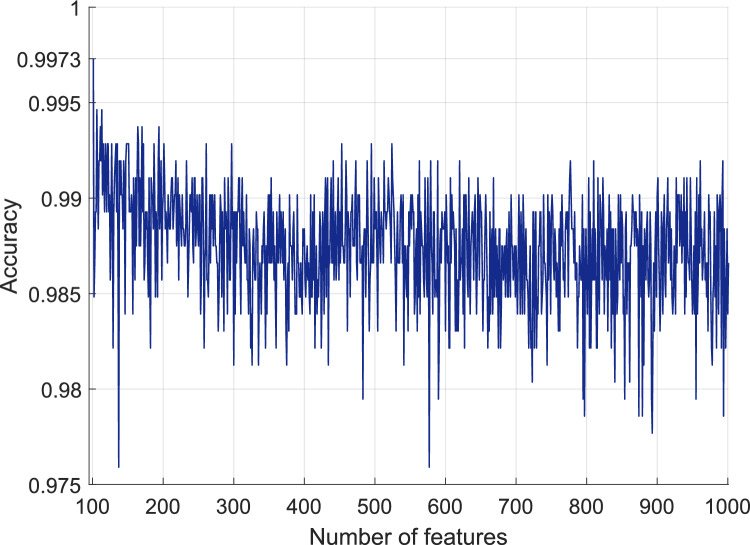
Classification accuracies of the selected feature vectors with various sizes

In our deep FE approach, we selected the most informative 100 features out of the initially generated 38,400 features. To accomplish this, we employed iterative NCA on the generated feature set. The range of iteration was defined to include 100–1000 features, allowing for the computation of classification accuracies for the selected 901 feature vectors. These comprehensive results, delineating the classification performance across different feature vector sizes, are presented in Fig. 8.

In Fig. 8, we present the classification accuracies obtained through the utilization of an SVM classifier with tenfold CV on the test image dataset. It is evident from Fig. 8 that the highest classification accuracy was achieved when employing the selected 100 features.

**Fig. 9:**
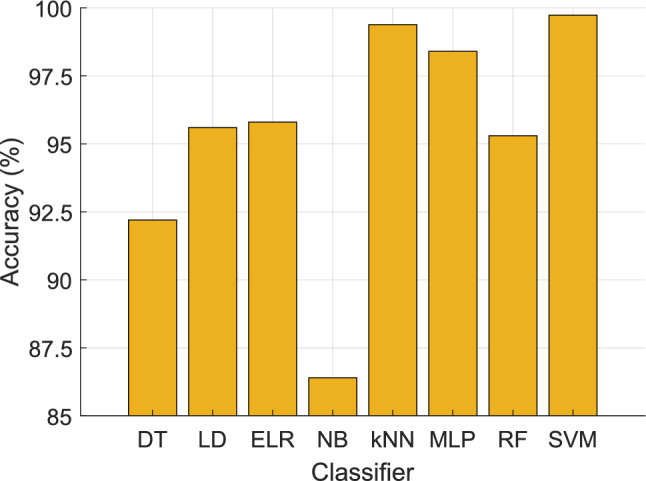
Comparisons of accuracies obtained by various classifiers

In our deep FE approach, the SVM classifier was chosen as the primary classifier due to its exceptional performance among the tested classifiers. Consequently, we exclusively employed SVM as the classifier in our model. To demonstrate the superiority of the SVM (Vapnik 1998), we compared its classification accuracy with conventional classifiers, including decision tree (DT) (Safavian and Landgrebe 1991), linear discriminant (LD) (Zhao et al. 1998), efficient logistic regression (ELR) (Tsangaratos and Ilia 2016), naïve Bayes (NB) (Ng and Jordan 2002), kNN (Maillo et al. 2017), multi-layer perceptron (MLP) (Biswas and Mia 2015), random forest (RF) (Pal 2005), and SVM. The classification accuracies of these classifiers are illustrated in Fig. 9, providing a comprehensive overview of their relative performance.

As illustrated in Fig. 9, among the eight tested classifiers, the SVM classifier exhibits the highest accuracy, achieving an impressive 99.73% accuracy. The kNN classifier follows closely as the second-best performer, with an accuracy of 99.38%.

**Fig. 10:**
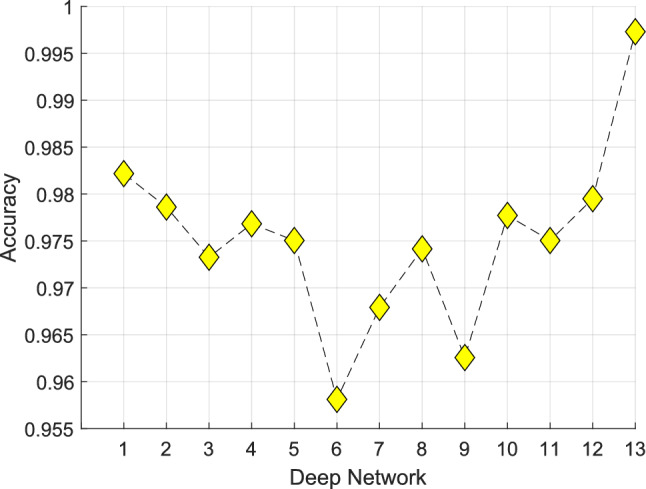
Classification accuracies for various deep networks

We have compared the performance of our approach with other deep-learning models such as (1) DenseNet201 (Huang et al. 2017), (2) ResNet50 (He et al. 2016), (3) MobileNetV2 (Sandler et al. 2018), (4) DarkNet53 (Redmon and Farhadi 2017), (5) ShuffleNet, (Zhang et al. 2018) (6) NasNetMobile (Zoph et al. 2018), (7) InceptionV3 (Szegedy et al. 2016), (8) InceptionResNetV2 (Szegedy et al. 2017), (9) GoogLeNet (Szegedy et al. 2013), (10) AlexNet (Krizhevsky et al. 2012), (11) VGG19 (Simonyan and Zisserman 2014), and (12) SqueezeNet (Iandola et al. 2016). We employed these networks to create a FE approach to select the most informative 100 features. The obtained test accuracies are depicted in Fig. 10, highlighting the superior performance of our proposed BrainNeXt (13th CNN in Fig. 10).

We compared our proposed BrainNeXt model with 12 other CNN-based models. As shown in Fig. 10, the DenseNet201-based exemplar deep FEX model achieved an accuracy of 98.22%, making it the second highest-performing network in Fig. 10. However, our proposed BrainNeXt-based model obtained the highest classification accuracy of 99.73%. Furthermore, we extended our evaluation by comparing the results of our proposed BrainNeXt-based model with other state-of-the-art models. The comparative outcomes are tabulated in Table 6, offering a concise summary of the performance comparison between our model and the leading models in biomedical image classification.

**Table 6:** Comparisons with other MRI classification models

Author(s)	Aim/number of classes	Method	Validation	Performance (%)
Payares-Garcia et al. (2023)	Neurodegenerative disease detection (AD, MCI, PD, MS, Healthy)/ Five class problem	Spatially informed Bayesian neural network, custom-designed CNN	Holdout validation (70:15:15)	Acc. = 83.0 Sen. = 85.0 Spe. = 81.0 F1. = 82.0
Tatli et al. (2024)	MS disease detection (MS, myelitis, control)/ Three class problem	Neighborhood component analysis, CNN	tenfold CV	Acc. = 97.63 Pre. = 97.23 F1. = 97.23
Acar et al. (2022)	MS disease detection/ Two class problem	Data augmentation, Custom designed CNN	fivefold CV	Acc. = 98.0 Sen. = 97.9 Spe. = 98.3 Pre. = 98.2
Balasundram et al. (2023)	AD severity detection (Non-demented, Mildly demented, very mildly demented, Moderate demented)/ Four class problem	Custom designed CNN	Holdout validation (80:20)	Acc. = 94.10 Pre. = 95.65 Rec. = 91.66 F1. = 93.61
El-Geneedy et al. (2023)	AD severity detection (Non-demented, Mildly demented, very mildly demented, Moderate demented)/ Four class problem	Data augmentation, Custom designed CNN	Holdout validation (80:20)	Acc. = 99.68 Sen. = 100 Spe. = 100 Auc. = 100
Abbas et al. (2023)	AD detection/ Two class problem	Jacobian map feed CNN	15-fold CV	Acc. = 94.20 Sen. = 94.64 Spe. = 93.75 Auc. = 96.66
Hussain et al. (2020)	AD detection/ Two class	Custom designed CNN	Holdout validation (80:20)	Acc. = 97.75 Auc. = 99.21
Our method	AD, MS, CI, control detection/ Four class problem	Custom-designed CNN (BrainNeXt), deep FEX from BrainNeXt, FS with NCA and SVM	tenfold CV	Acc. = 99.73 Pre. = 99.75 Rec. = 99.55 F1. = 99.65

As shown in Table 6, most of the studies are focused on DL. These methods have high computational complexity, but they achieve high classification success (Marwa et al. 2023). In our study, FEX is achieved by using our architecture. In this way, a lightweight approach has been obtained compared to the literature. Literature studies generally focus on disease level (Balasundaram et al. 2023) or disease detection (binary classification) (Acar et al. 2022). In our paper, a new multi-class dataset was collected and classified. Garcia's approach (Payares‐Garcia et al. 2023) is similar to our study in terms of the dataset. However, some neurodegenerative diseases (Alzheimer, Mild cognitive impairment, PD, and MS) are considered in their study. In our dataset, in addition to neurodegenerative diseases (AD and MS), CI is also included and there is no such dataset in the literature. Moreover, when the accuracy values of the studies given in Table 6 are analyzed, our model is prominent. The model developed in this research surpassed 99% in all metric values. These results showcase the superiority of the suggested method.

Table 6 compares the binary and multi-class tasks. The binary classification tasks (e.g., detecting a specific disease or distinguishing between sick and healthy cases) are less complex due to two classes. The multi-class tasks, such as our 4-class classification presents additional challenges as it involves various diseases and healthy controls. The proposed BrainNeXt and BrainNeXt-based deep FE models perform better for 4-class task compared to the binary classification tasks, highlighting the high classification capabilities of the proposed models.

BrainNeXt has the ability to extract clinically significant features using the modified ConvNeXt structure and inter-layer concatenation functionality.

**Fig. 11:**
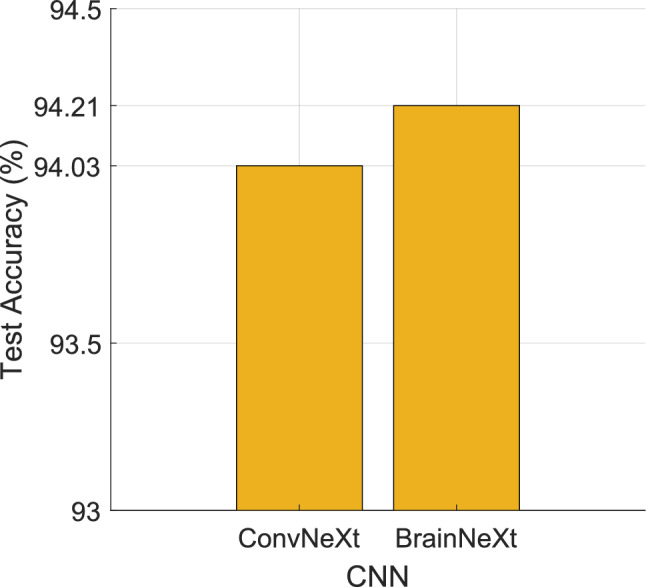
Test classification accuracies obtained for ConvNeXt and BrainNeXt models using the collected dataset

In the BrainNeXt-based deep FE approach, the proposed BrainNeXt architecture, combined with patch-based (transformer-like) FE, is able to extract discriminative features across all classes. The inclusion of chronic ischemia with neurodegenerative diseases (AD and MS) provides a dataset that is more representative of real-world scenarios. Advanced FS techniques, such as NCA assists in the extraction of salient features and boost the classification performance. Additionally, the recommended model was compared to the base version of ConvNeXt, and the test classification accuracies of these models are shown in Fig. 11.

Figure 11 indicates that BrainNeXt achieved higher test classification performance than ConvNeXt due to the use of concatenation and leaky ReLU functions.

The obtained/calculated results highlight the superior classification performance of our proposed approach compared to other state-of-the-art models.

Key points of our research are outlined below:

- We acquired a new MRI dataset encompassing AD, chronic ischemia, MS, and control cases. We have made this dataset publicly available, aiming to contribute to the field of biomedical image classification.
- A lightweight CNN model, BrainNeXt, was introduced, boasting a mere 8.9 million trainable parameters.
- Inspiring the advantages of ConvNeXt and vision transformers (ViT), we proposed both the BrainNeXt model and an exemplar deep FE approach.
- Our proposed models achieved high test accuracies of 94.21% and 99.73%, respectively.
- Notably, we did not rely on any fine-tuning operations to attain these high classification performances.
- The presented BrainNeXt model exhibited the lowest accuracy for the chronic ischemia class, likely due to the limited availability of MRI samples for this class. Conversely, the control class yielded the highest accuracy, likely due to its larger sample size.

Demerits:

- The potential to test the proposed BrainNeXt model on larger and more diverse MRI datasets to further validate its performance.
- The possibility of exploring different versions of the BrainNeXt model, such as nano, femto, tiny, base, and large, to assess their respective capabilities and scalability.

Future works:

- We intend to test our developed model by collecting more diverse images belonging to various races and severity of classes.
- Various versions (Nano to large) of the proposed BrainNeXt model can be developed for different applications.
- We may have to modify the proposed model using patches to detect classes with unclear or overlapping symptoms.
- A novel XAI interface can be employed to assist clinicians in confirming their findings.
- Our developed BrainNeXt model can be used to as a next-generation intelligent radiologist assistant for clinicians.

## Conclusions

Our research has demonstrated the superiority of our proposed approach in achieving high-performance classification results for MRI image analysis. Through the development of the BrainNeXt model, we have introduced a lightweight CNN architecture with a compact parameter count of only 8.9 million. This innovative model capitalizes on the strengths of ConvNeXt and ViT, enabling efficient and effective FEX for accurate classification.

Our presented BrainNeXt reached 94.21% test accuracy and our BrainNeXt-based deep FE approach yielded 99.73% on the test dataset. The computed experimental result underscored the high classification performance of the presented BrainNeXt. Especially, these outstanding results were obtained without applying fine-tuning operations to underscore the high MRI classification capability of our proposed models.

Our proposals have contributed to the advancement of biomedical image classification and serve as a foundation for future investigations in this domain since we designed a new network by using the structure of the CNNs for the 2020s.

For future research, expanding the evaluation of the BrainNeXt model on larger and more diverse MRI datasets will enable us to assess its performance robustness and generalization capabilities. Additionally, exploring various versions of the BrainNeXt model, including nano, femto, tiny, base, and large variants, can further elucidate the model's scalability and potential for broader application domains. Moreover, patch-based models like transformers and ConvMixer can be proposed to increase validation scores.

## Data Availability

The data presented in this study are available on request from the corresponding author. The data are not publicly available due to restrictions regarding the Ethical Committee Institution.
